# Duplication of Vertebral Pedicles Associated with a Thoracic Burst Fracture Resulting in Spinal Cord Compression: A Case Report

**DOI:** 10.7759/cureus.3820

**Published:** 2019-01-03

**Authors:** Ryan Johnson, Paul J Gustin, Jason M Seibly

**Affiliations:** 1 Neurosurgery, Advocate Bromenn Medical Center, Normal, USA; 2 Neurosurgery, Central Illinois Neuroscience Foundation, Bloomington, USA

**Keywords:** pedicle duplication, burst fracture, kyphosis

## Abstract

Vertebral body fractures are well-known sources of axial back pain with the potential to cause neurological deficits. Duplication of a component of the vertebral column is a rare phenomenon; however, vertebral pedicle duplication is an unreported phenomenon, and has not been reported in association with a vertebral burst fracture and kyphotic deformity. We present a unique case of vertebral pedicle duplication in association with a T11 vertebral burst fracture in a 72-year-old female. In addition to her risk factors, it is the belief of the authors that the anatomic anomaly increased the segmental kyphosis at the level of the T11 vertebral body, thereby increasing the axial load on that segment, and increasing the risk for fracture.

## Introduction

Vertebral body fractures are a common pathology, especially in postmenopausal and osteoporotic women, and are a common source of axial back pain. Denis and colleagues devised a three-column system to help categorize the stability of these fractures [1]. The anterior column includes the anterior longitudinal ligament, anterior half of the vertebral body, and anterior half of the intervertebral disc. The middle column includes the posterior half of the vertebral body, the posterior half of the intervertebral disc, and the posterior longitudinal ligament. The posterior column includes the posterior osteoligamentous structures that envelope the spinal canal [1]. Typically, these fractures manifest as compression fractures, which indicates an anterior column failure secondary to intolerable axial loading on the vertebral body. In more severe cases, the anterior and middle columns of the vertebral body will fail, referred to as burst fractures, which may result in bony retropulsion into the spinal canal, thereby potentially compressing the spinal cord or spinal nerve roots. Neurological deficits can ensue from this compression, which commonly will require surgical decompression and stabilization. Risk factors for these fractures include osteoporosis, trauma, chronic kidney disease, prolonged immobility, obesity, vitamin D deficiency, and bony malignancy. We present here a unique case that identifies a patient with multiple risk factors for a vertebral body fracture, but also has an anatomical anomaly that may have contributed to an exaggerated segmental kyphosis, and unequal axial load sharing to that vertebral body; thereby, increasing the potential for compression or burst fracture. To our knowledge, this is the first case of a duplicated vertebral pedicle in association with a vertebral burst fracture.

## Case presentation

A 72-year-old Caucasian female with a significant past medical history that includes stage IV chronic kidney disease, insulin-dependent diabetes mellitus, hypertension, peripheral artery disease with bilateral below-knee amputations, and atrial fibrillation presented to the emergency department with a chief complaint of a four-month history of progressive lower back pain that radiated into both buttocks and her anterior and posterior thighs. Additionally, over the prior two weeks, she began to have episodes of urinary incontinence and noticed paresthesia in her bilateral medial thighs, stating the left side was worse than the right. She denied any recent trauma, although she did elicit a history of falling out of her bed eight months prior to admission. She is wheelchair bound, and requires assistance for all her activities of daily living. Her physical examination was remarkable for morbid obesity, with a body mass index (BMI) of 43.5 kg/m2, and bilateral below-knee amputations. Neurologically, her examination was unremarkable other than diminished light touch sensation to bilateral medial thighs.

**Figure 1:**
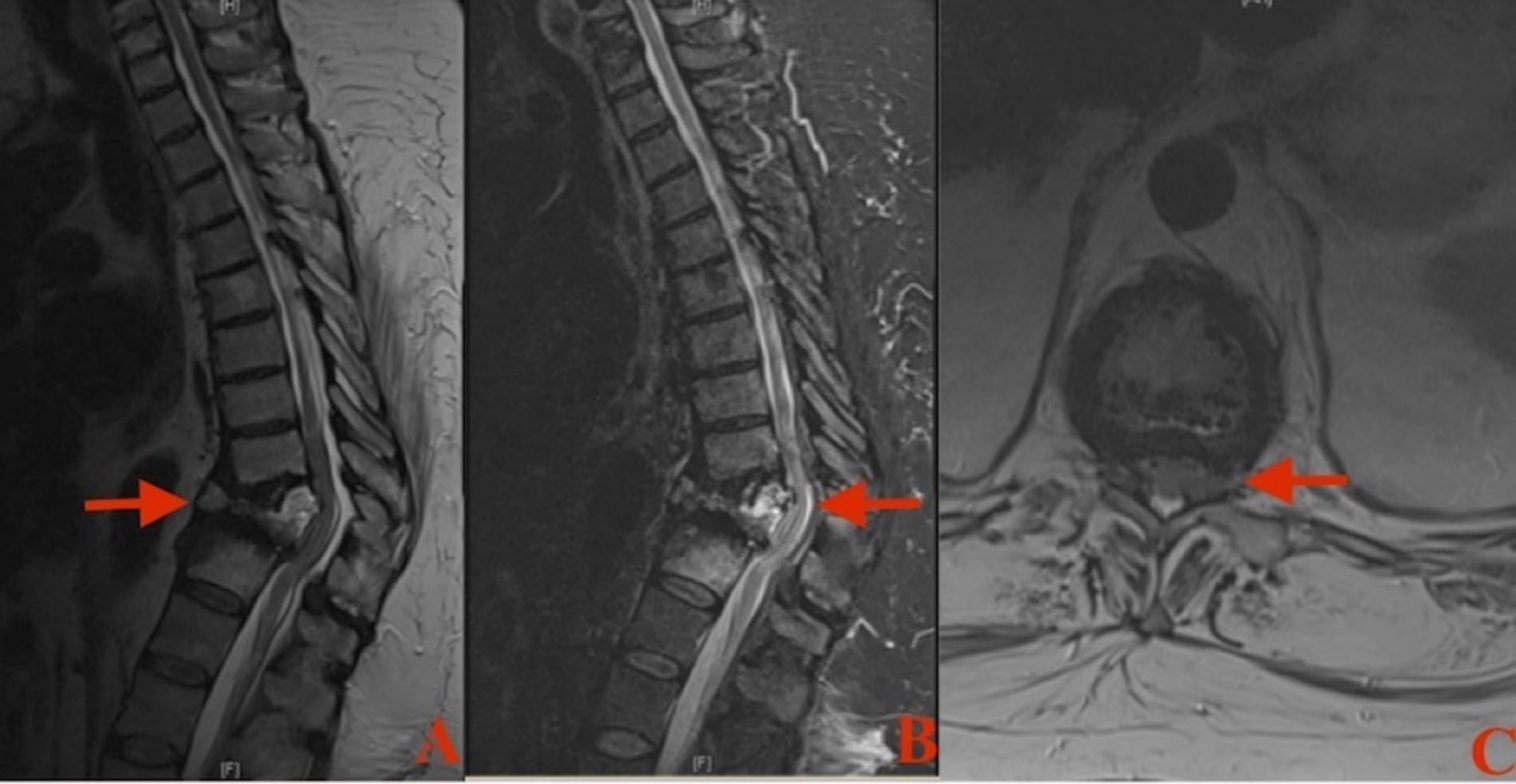
Magnetic resonance imaging (MRI) of the thoracic spine A: Sagittal T2-weighted image demonstrating a T11 burst fracture (arrow) with posterior bony retropulsion and spinal cord compression. B: Sagittal T2-Short Tau Inversion Recovery (STIR) demonstrating areas of hyperintensity within the T11 vertebral body consistent with acute inflammation and intramedullary hyperintensity at T11/T12 consistent with myelomalacia (arrow). C: Axial T2-weighted image at T11/T12 intervertebral disc demonstrating severe spinal canal stenosis with cord compression anteriorly (arrow).

**Figure 2:**
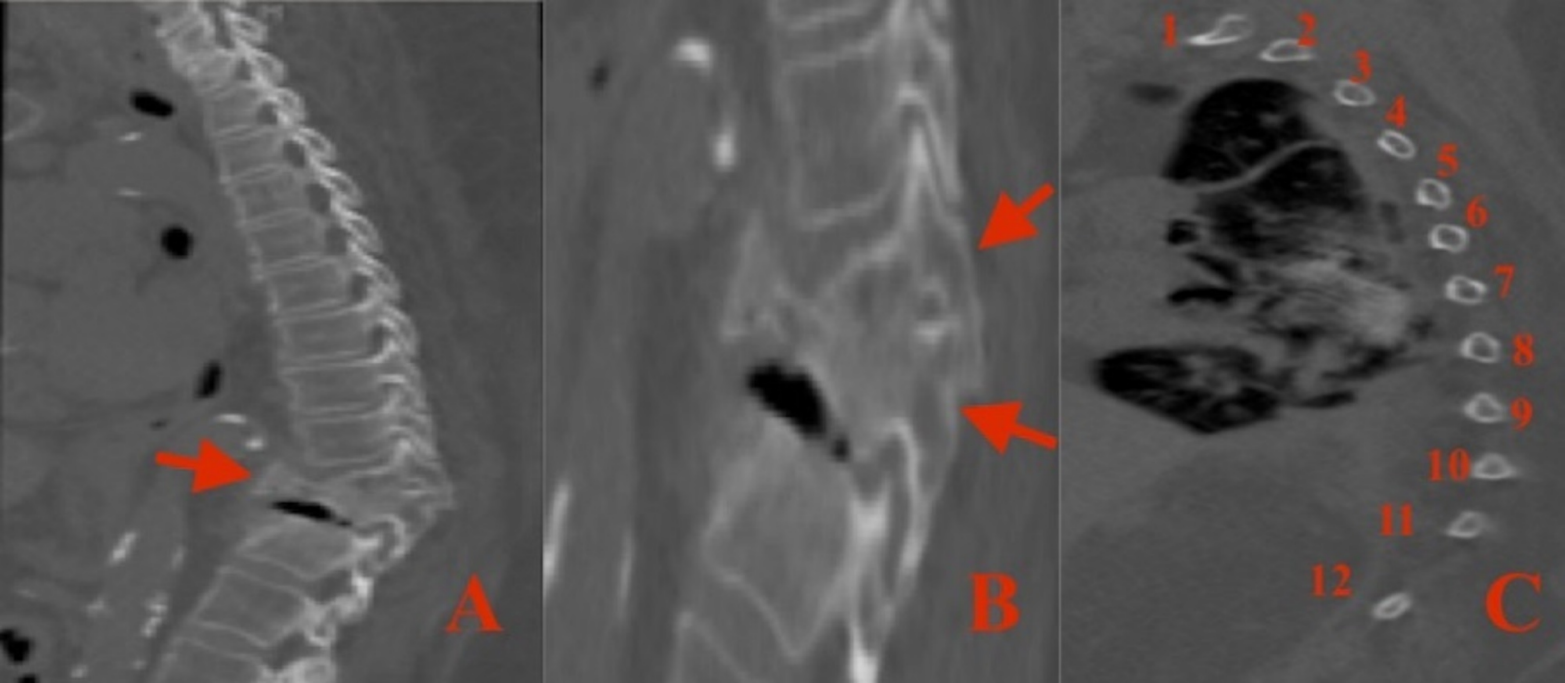
Computed tomography (CT) of the thoracic spine A: Sagittal view demonstrating a T11 burst fracture (arrow) with posterior bony retropulsion, and vacuum disc phenomenon of the T11/T12 intervertebral disc space from advanced degeneration. B: Sagittal view with zoom focused on the T11 vertebral body showing duplication of the vertebral pedicles entering the T11 vertebral body in vertical alignment (arrows). C: Sagittal view utilized for accurate counting of the 12 thoracic ribs, and for verification of their corresponding vertebral body.pedicles entering the T11 vertebral body in vertical alignment (arrows).

Magnetic resonance imaging (MRI) of the thoracic and lumbar spine was ordered that identified a subacute T11 burst fracture with bony retropulsion resulting in central canal stenosis, and severe cord compression at the T11/T12 interspace with associated myelomalacia (Figures 1A-1C). Additionally, there was a segmental kyphotic deformity associated with this fracture. To better delineate the bony anatomy, a computed tomography (CT) of the thoracic spine was obtained. In addition to the T11 burst fracture and vacuum disc phenomenon from advanced degeneration at the T11/T12 intervertebral disc, an anatomical anomaly was identified. Duplication of the T11 vertebral pedicles in vertical alignment was demonstrated on axial and sagittal views (Figures 2A-2B). To confirm this to be an actual anomaly and not an auto-fusion of adjacent vertebral bodies from a severe compression deformity, the ribs were counted and followed to their respective vertebral body (Figure 2C). The total rib count was 12 on each side, and was associated with the correct vertebral body.

To help alleviate the patient’s severe back pain, bilateral leg pain, and paresthesia, and to diminish the risk of permanent urinary and fecal incontinence, the patient underwent a T10 to T12 laminectomy with posterior instrumented fusion via pedicle screws inserted into T9, T10, T12, and L1 using stealth neuronavigation. Intraoperatively, the anomaly was visualized as an elongated T11 lamina with two pedicle screw entry points on each side into the T11 vertebral body identified with the stealth navigation probe. The laminectomy and instrumented fusion were performed in standard fashion. The patient tolerated the procedure well, and there were no intraoperative complications.

**Figure 3:**
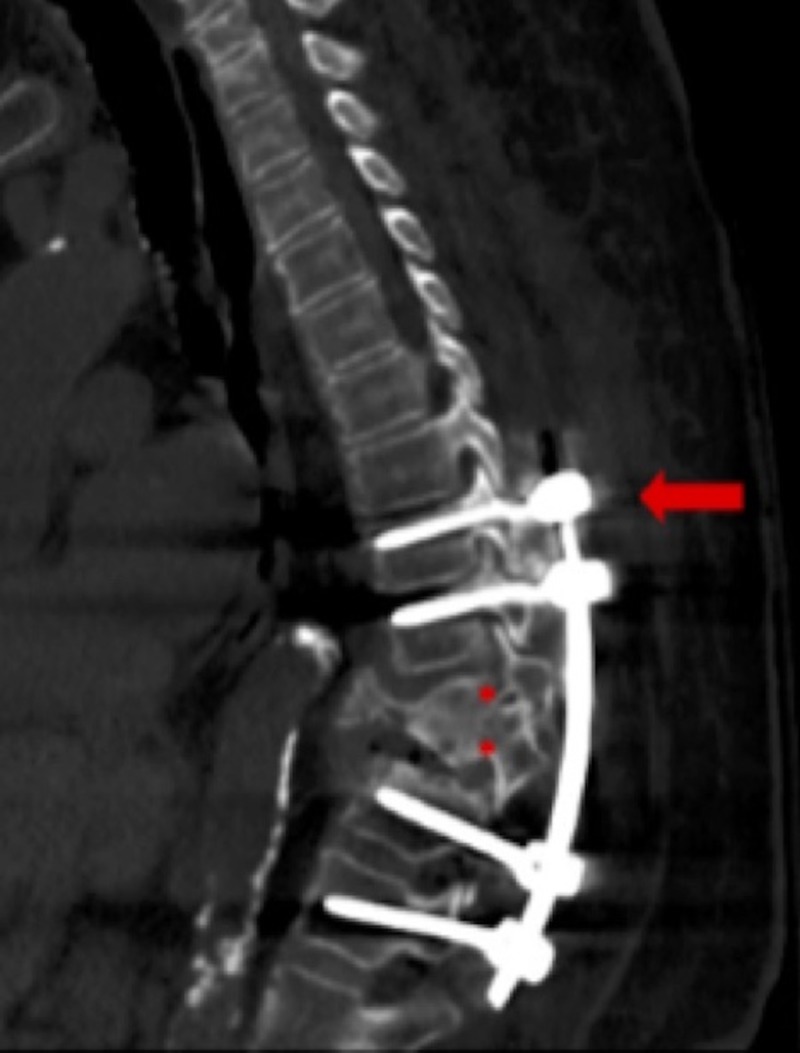
Computed tomography (CT) of the chest two months postoperatively Sagittal view demonstrating proper placement of pedicle screws at T9 (arrow), T10, T12, and L1. The duplicated T11 pedicle is also seen on this sagittal projection (asterisks).

Postoperatively, the patient had improvement in her bilateral leg pain and paresthesia. Her Jackson-Pratt drains were removed on postoperative day two, at which time she was deemed stable for discharge to a rehabilitation facility. She was discharged to a skilled nursing facility on postoperative day six. She was readmitted to the hospital two months after surgery for hospital-acquired pneumonia. At that time, a CT of the chest was performed that showed the hardware to be in the proper position as well as help re-identify the T11 duplicated pedicle (Figure 3).

## Discussion

Developmental anomalies of the spine are not uncommon and are well documented in the medical literature. The embryology of the spine and the malformations associated with dysfunctional spinal development is beyond the scope of this paper; however, the authors would like to refer the readers to an excellent review written by Chaturvedi et al. in 2018 for more information [2]. Duplication of a vertebral pedicle, to our knowledge, has not been documented as a developmental anomaly, nor has it been documented in association with a vertebral burst fracture. The paper cited above does discuss pedicle malformations, but limits the discussion to short pedicles associated with symptomatic spinal stenosis, and pedicle agenesis mistaken for an acute trauma [2]. Duplications of spinal lamina have been described in the literature, in which the duplicated lamina will form below another lamina, leading to spinal cord compression [3-4]. Another case report details a unique case of C7 spinous process duplication that was identified post-mortem while teaching medical students through cadaveric studies [5]. There have also been several case reports that detail patients with entire spine duplications of various segments, some which were found incidentally while imaging for unrelated medical conditions, and others who varied in symptoms suggestive of a tethered cord syndrome [6-7].

This small body of literature demonstrates the variability in spinal duplication, and despite its rarity in current literature, vertebral pedicle duplication should be recognized as a possible anatomic anomaly. The etiology and clinical significance of this anomaly cannot be ascertained from a single case report. While the literature supports congenital causes for spinal duplications, the authors do not have radiographic imaging to document the existence of the anomaly prior to this presentation. It is possible the patient suffered a compression deformity from her fall eight months prior; however, the authors do not feel this explains the existence of duplicated pedicles bilaterally. The remote trauma could, theoretically, have fractured both T11 vertebral pedicles allowing for the appearance of duplicated pedicles; however, the symmetrical location of the duplicated pedicles bilaterally would make a traumatic etiology less likely. The concept of vertebra plana, and auto-fusion to an adjacent vertebra was investigated by counting the number of ribs and their articulation with their respective vertebra. As stated above, there were 12 total ribs on both sides, each of which corresponded to the correct thoracic vertebra. In the same respect that pedicle subtraction and Smith-Petersen osteotomies increase segmental lordosis, can a duplicated vertebral pedicle impose a segmental kyphosis, and shift the vertebral axis for physiologic loading? In addition to the patient’s risk factors for bony demineralization (prolonged immobility, chronic kidney disease with probable vitamin D deficiency, morbid obesity, and suspected osteoporosis), this shifted axis could have increased the risk of anterior column failure in the T11 vertebral body. Worsening kyphosis would then further shift the axis of physiologic loading, and result in middle column failure with eventual bony retropulsion into the spinal canal with spinal cord compression. The authors believe this question and scenario to be plausible, but further study of this anomaly is needed to verify this assertion.

## Conclusions

Developmental anomalies of the spine are increasingly being reported in the medical literature. These anomalies can present with a wide range of symptoms, and be a cause of morbidity in the cases of spinal cord or spinal nerve root compression. We reported the first known case of vertebral pedicle duplication in a patient with an unstable thoracic burst fracture. Treating physicians and surgeons must continue to reinforce their understanding of normal spine anatomy, as well as educate themselves on the increasing amounts of anatomic variations in order to provide optimal patient care.
